# A Gig mHealth Economy Framework: Scoping Review of Internet Publications

**DOI:** 10.2196/14213

**Published:** 2020-01-15

**Authors:** Fahad Alanezi, Turki Alanzi

**Affiliations:** 1 Community College Imam Abdulrahman Bin Faisal University Damamm Saudi Arabia; 2 Health Information Management and Technology Department, College of Public Health Imam Abdulrahman Bin Faisal University Dammam Saudi Arabia

**Keywords:** gig economy, gigs, mHealth, sharing economy, gig mHealth

## Abstract

**Background:**

The gig economy (characterized by short-term contracts rather than being a full-time employee in an organization) is one of the most recent and important tendencies that have expanded through the global economic market thanks to advances in internet and communication technologies. Similarly, mobile health (mHealth) technologies have also evolved rapidly with the development of the internet and mobile apps, attracting attention globally for their health care benefits.

**Objective:**

This study aimed to propose an integration of mHealth within the framework of the gig economy that leads to a new dimension of health care services and the proposal of a new term: gig mHealth.

**Methods:**

A review and systematic search of articles, books, and opinions that allowed for answering the research questions were executed through the internet. In this sense, the concept of the gig economy and examples, advantages and disadvantages, were reviewed. Similarly, the general characteristics of mHealth technologies were revised. In addition, the role of technology in supporting the development of the gig economy and mHealth technologies and the interactions between them were investigated.

**Results:**

The findings suggested that the gig economy is characterized by its flexibility in working hours, on-demand work, free agents, freelancing, freedom in the choice of work, and independent contracts. In addition, an analysis of an mHealth system indicated that it was composed of patients, specialists, nurses, and database administrators. In this system, patients and specialists or nurses are connected to cloud services for the transmission of data and medical information through a mobile app. Here, the administrators update the database and app features, among other technical tasks. Conversely, a general structure of an integrated gig mHealth system was developed. In this structure, the mHealth care services and the mHealth care activities were incorporated into a gig economy model. In addition, a practical example of an integrated view of a gig economy app in mHealth that illustrates the interaction between the patients (consumers) and providers (partners) of mHealth care services, mHealth care activities, health care professionals, and individual contractors was presented. The consumers and providers were interconnected with the health care company, brand, or firm through digital means using a mobile app or Windows platforms.

**Conclusions:**

The analysis carried out in this study suggested the possibility of integrating mHealth within the framework of the gig economy enhancing health care service delivery and the management of health care activities. The following 4 major areas of apps proposed in the mHealth framework that can catalyze the operations using the features of the gig economy were sharing/renting medical and diagnostic equipment and resources, on-demand appointments/self-health management, on-demand health care services, and assigning health care activities/gigs to individual contractors. This integration leads to a new dimension for health care services and the proposal of a new term: gig mHealth.

## Introduction

### Background

A major changing trend has been observed in the work culture during the last few years. The majority of millennials and employees are shifting toward a working model that is identified by freedom in working hours, choice of work, freelancing, and small tasks (gigs), characterized by short-term contracts rather than being a full-time employee in an organization. This changing tendency is known as a gig economy, and it has been increasing rapidly. It was calculated that there were more than 5 million gig workers in the United Kingdom [1]. In addition, it was estimated that approximately 34% of the working people in the United States is the portion of the gig economy population, which is expected to reach 43% by the year 2020 [2]. This changing trend is observed across various countries, and it is impacting the work culture of organizations, products, and delivery services through the various sectors of the global economy [3]. Extensive research is needed to be able to determine the benefits of such an economy. However, the benefits, flexibility, and freedom of working in a gig economy are attracting the attention of various organizations, millennials, and researchers to investigate the possibility of integrating the concept into various systems. Previously, this conception has been applied successfully in several companies around the world: Uber, Turo, and Upwork [4-6]. Uber is a car rental company, Turo is a sharing car market place, and Upwork is a mobile platform for hiring freelancers in different fields of universal knowledge.

In relation to health care, we found that some companies such as Nomad, Enzyme, and Medely have used some ideas from the gig economy to hire doctors and nurses to perform independent and short-term jobs in hospitals [7-9]. However, in the review of the literature, no company was found that systematically applied the principles of gig economy in mHealth that integrate the use of the internet, medical sensors, mobile computing, and communication technologies for managing and delivering health care access by diverse workforces and activities [10-12].

Linking mHealth and gig economy features may enhance the performance features of mHealth and ensure effective management and delivery of health care services. Considering mHealth as a point of focus, the aim of this study was to propose the possibility of integrating mHealth into the gig economy features for the effective management of health care services. This option conducts to a new dimension for health care services and to the proposition of a new term: gig mHealth.

### Gig Economy

The gig economy is a relatively new term that has attracted the attention of the world because of its accessibility to employment opportunities and to the various legal, ethical, and business complications arising from it. It has been defined from various perspectives, considering the factors of influence. A *gig* usually refers to a job or task, which often has a short-term connection with a particular business. The workers in such a scenario are employed in a specific task assigned for a particular period [13].

The gig economy also refers to an on-demand economy with independent work arising out of choice and necessity according to the McKinsey Global Institute Report, in which the independent workers are classified into 4 segments: free agents, casual agents, reluctant workers, and financially strapped workers [3]. According to this report, 162 million people who represent 20% to 30% of the working population in Europe and the United States are involved in the gig economy. This information reflects the diversity and growth of the gig economy.

Similarly, the gig economy is denoted as a sharing economy where the operational tasks in the business are divided into smaller tasks that are assigned to independent contractors who cannot be considered as employees. The business aim is to reduce the infrastructure and resource costs by sharing the work with independent workers at lower wages compared with an employee’s wages. It was calculated that the gig economy was worth US $26 billion in 2015 [14].

Another approach to defining the gig economy is identified from the perspective of the types of work carried out: crowdwork and work on demand via an app [15]. In *crowdwork*, a series of tasks, which have to be completed over Web-based platforms, are outsourced to a group of individuals or organizations, and “work-on-demand via app” is a modality in which traditional working activities such as cleaning, babysitting, clerical work, software development, and lawyers are accessed directly by app users [15].

In general, the features of a gig economy consider work on demand, free agents, freelancing, independent work, flexibility in working hours, freedom of choice of work, and independent contracts.

Although the concept of the gig economy is seen by a few as a boon for the employed and unemployed, others have raised various concerns over its benefits. Therefore, there is a need for a clear understanding of its nature, implications, advantages, and underlying issues and concerns.

### Role of Technology in Enhancing the Gig Economy

The advances in the internet and communication technologies are considered to be one of the most effective means through which the gig economy is developing. This has enabled the organizations to restructure their operational activities in a way that the greater share of the work is assigned to the individuals who are not the employees of the organizations, thereby reducing the operational costs. The new technologies also make it easier to organize the workforce based on the project or specific skills on a short-term contractual basis [13,16]. Unlike the days before the internet, technology has enabled organizations to find the workforce desired, pay them small amounts, and fire them when they are not needed without any obligations. Similarly, the individuals can accept any contract of work for the time they wish and opt out without obligations. In addition, individuals from rural and remote areas can have access to the jobs at a national or an international level using internet technologies in the gig economy [17].

Developments in smartphone technology have identified new ways for accessing gig work, reshaping the market space, and providing new ways to access this market by the consumers and workers. Its ability to reach remote areas and impact a large group of workers and their livelihoods through the smartphone and other technology apps have made the gig economy one of the most preferred ways of earnings [18]. With the technology support in gig economy, new terms have been proposed such as digital labor, digital workforce, and Web-based freelancers and the need has been felt for redefining the labor policies in light of the growing size of the gig economy [19]. Furthermore, the increasing social presence of people on the Web-based platform and new business promotion techniques through social networking technologies have paved the way for advancing gig economy deployment in various industries, including transportation, hospitality, traditional services such as plumbing, tuitions, rentals, and consultations, and other businesses.

### Advantages and Risks/Issues in the Gig Economy

The gig economy can significantly improve the overall employment rate and enhances the freedom and choice of work because of which it is seen as a boon to the new entrepreneur generation, as shown in the study by Burtch et al [20]. It helps the entrepreneurs to minimize the investment costs and gain the maximum output by streamlining the operations across the individual contractors at lower wages [21]. Extreme flexibility in the gig economy has worked as an advantage for the workers who are interested in having freedom in working hours, working location, and finances. In addition, computerization and globalization have enhanced the gig economy with potential benefits for deployment [22]. The gig economy also offers a platform for older people who have retired from their jobs, offering more flexibility to work. In a study conducted by Zurich UK, an insurance company, it was found that over one-third of the people older than 55 years moved to the gig economy for temporary jobs to ease the transition into retirement [23]. The gig economy can be beneficial to consumers as they can effectively and directly access various services for low costs without mediators or share costs. For example, Uber allows drivers with personal vehicles to be part of the force, helping the company to minimize the investment costs while providing benefits to the gig worker or driver, and the concept of share ride on its app reduces the cost of a ride for the consumer [24]. Similarly, a recent start-up, *InCloudCounsel,* provides various legal services offered by a group of lawyers that can be accessed by the consumers without any intermediaries [25].

On the contrary, various issues and concerns have being raised over the gig economy. One of the major issues is the perspective in which an individual or independent contractor is observed by the business. They are not considered as employees by the organizations and are not benefited from a provident fund, pension scheme, insurance, and retirement policies. In addition, the minimum wages for independent workers are rarely met, and the bulk of business risk is shifted to the workers, making them vulnerable to many risks [21,22]. Another important observation is that with the evolution of contingent work in the gig economy, new employee-employer relationships have emerged, and a question to be considered is whether or not the legal frameworks governing the workforce have updated the system with these new relationships [26]. Referring to workers as independent contractors and not as employees benefits the organizations by avoiding additional costs. This has proved to be the litmus test for the courts to determine the status of the workers [27]. It has also been suggested that entrepreneurial activity may be reduced as the gig economy offers stable employment for the employed and unemployed [20]. As it is known, safeguarding the retirement plans and savings is one of the important aspects of any employment. However, only 16% of the gig workers have retirement plan schemes, whereas 52% of the employees have access to employer-sponsored retirement plans [28].

As the gig economy is relatively new and developing rapidly, it has to be observed through various case studies and through extensive research to investigate and address the various issues surrounding it.

### Review of Gig Economy Cases

There are various examples in which the gig economy has proved to be successful, effective, and efficient in delivering services and enhancing employment opportunities. To investigate the opportunities and issues that come with the application of the gig economy, some of the major cases such as Uber, Turo, and Upwork models are reviewed in the following sections.

#### Uber Model

Uber, a car rental company, is one of the first adopters of the gig economy, and perhaps the most widely considered example. It started in 2009 as a different alternative to common taxi services and has quickly grown to be one of the top multinational companies in the world, with an estimated worth of US $65 billion [4]. Uber is a mobile app that acts as a platform and a point of contact for the drivers and riders. Various services are offered on the platform, including premium and limousine rentals (Uber Black), less expensive rides with medium-range cars (Uber X and Uber Go), and economy services through Uber Taxi and delivery services such as food through Uber Eats. Both drivers and passengers are required to agree upon Uber’s terms and conditions on the app, which declares that both are independent contractors, are subject to ratings and reviews, and the services can be denied if the ratings fall below Uber’s threshold [4]. The app platform is simple and easy to use. The passengers can log in to the app and request a service for their location. An alert is raised with the nearby drivers on the Uber app, and the request can be accepted by the drivers. However, the drivers are not bound to accept all requests, giving them the freedom to work as per their convenience. If the passenger desires, the approximate fare is presented in the app before confirming a booking. Once the booking is confirmed, the driver picks up the passengers and drops them at the desired location given at the time of booking. The payment can be made through electronic wallets, cash, credit cards, or other means [29].

#### Turo Model

Turo is a Web-based and mobile app for car owners who do not wish to drive and can rent their cars for a day or more. It is a car-sharing marketplace, where the travelers can rent any car they want from a community of local car owners. It has its operations spread across more than 4500 cities and 300 airports with a fleet of more than 800 models. The owner’s average monthly earnings are estimated to be US $720 [5]. The users can sign up on the mobile app or on the website and need to accept the terms of service. The users can then search for the car according to their needs and interests and book the car. The charges for the daily rentals are given for each car, and the price may vary with the model and year of manufacture of the car. The rental price is finalized by Turo after assessing the condition of the car. Once booked, the owners have 8 hours to accept or reject the request. The users can either pick up or request delivery of the car and then return it after completing the trip. All the cars are insured, and Turo takes all safety measures before renting out. Similarly, the car owners can register and list their car on the Turo platform. They can respond to the requests made and can either deliver or give out the car and thus can earn while not using the cars [5].

#### Upwork Model

This company is one of the largest Web-based and mobile platforms for hiring freelancers in a wide range of operations including Web, mobile, software development, graphic/creative designing, administrative support operations, information technology and networking, writing work, sales and marketing, data sciences and analytics, translation, legal affairs, accounting and consulting, and other tasks. With an extensive workforce in more than 3500 skill areas, freelancers across the globe are earning more than US $1 billion every year through Upwork [6]. The platform provides an easy interface not only for hiring freelancers but also for freelancing. The people who want to hire freelancers can register and start posting jobs, which are analyzed by Upwork, and a short list of likely candidates is sent to the hirers. The hirers can then browse the freelancers’ profiles, review proposals, and schedule a chat before offering work. The hirers can send and receive files over the app in a secure environment and share feedback in real time. In addition, the payment process is simplified as Upwork delivers payments for freelancers in more than 170 countries through the effective global payment network, with invoicing and reporting capabilities [30]. The freelancers have the freedom to work on ideal projects and can ensure more success. A streamlined hiring process in Upwork ensures the projection of the right applicants for the job. Hourly/fixed price projects give more freedom to choose from the types of work by the freelancers. The service fee for freelancers is nominal, where 20% of the billing amount is charged for the transactions up to US $500; 10% for billings between US $500.01 and US $10,000; and 5% for billings that exceed US $10,000 [31].

These are the 3 major gig economy examples that have been growing rapidly. There are various other examples in various sectors where the gig economy has proved to be successful. Therefore, the gig economy can be streamlined into different sectors to promote easy delivery of services with a sharing economy and enhanced operational efficiency.

### Gig Economy and Health Care

The health care systems have already started to attempt the gig economy, which can be observed in the temporary hiring of nurses, doctors, technicians, and other clinical specialists [32].

This trend is, among other causes, because of the increase in the costs of medical attention, the shortage of medical personnel, and the fact that many health care professionals find that the gig economy offers them more versatile and lucrative independent jobs [32,33]. One of the objectives of the gig economy in institutions dedicated to health care is to reduce labor costs, optimize the needs of medical personnel, and at the same time provide excellent medical care to patients. In general, the gig economy can offer various benefits to the health care systems [33].

On the contrary, under this modality of the economy, health systems can hire temporary professionals when they need them, and the professionals can obtain the best work opportunities adapted to their interest [32].

For example, Nomad is a Web-based platform through which independent doctors negotiate their contracts with hospitals [7]. Under this modality, both the hospital and the doctor achieve what they want, they save money, and Nomad receives a commission to facilitate the process [7]. Other platforms connecting doctors to hospitals and other health care facilities are Enzyme and Medely [8,9].

In addition, the British government has proposed to design an app for England’s National Health Service that delivers on-demand health care based on the gig economy style [34].

### Mobile Health

mHealth is a terminology used in the application of mobile/communication and internet technologies for the delivery of health care [10,35]. It is defined as “emerging mobile communications and network technologies for healthcare systems” [10]. It can also be described as the integration of mobile computing, communications technologies, electronics, sensors, and medical services to provide mHealth care applications [12,36,37]. In general, the definition of mHealth varies according to the areas of application, the services involved, and the technologies used. The applications of mHealth technology have been on a rapid increase in recent years, attributing to the enormous increase in the use of smartphone and internet technologies across the globe [11,35]. Various authors have identified that mHealth can have a significant impact on the management and delivery of health care services by reducing the operational costs and increasing operational efficiency [38,39]. In addition, it is convenient to point out that despite the benefits provided by the use of mHealth technologies to improve the health care delivery and motivate patients for self-care, there are risks related to the privacy and security of patient data when these technologies are used [35,40].

In general, a common mHealth structure includes 3 main actors: patients, specialists/nurses, and database administrators [11]. The patients self-handle their diseases remotely utilizing a mobile app that is linked to the cloud services for the transfer of data and information. The smart app can also be integrated (using Bluetooth, Wi-Fi, or any other connection) with biosensors or medical devices such as blood pressure monitors, glucose sensors, or other instruments. The data from the biosensors located on the patients’ body can be sent to servers on the cloud using the internet connection. Then, the specialists/nurses connected to these servers on the cloud examine the collected data and return medical information based on the diagnosis results/queries placed by the patients. The administrators carry out all the technical tasks such as updating the database/app features, data entry work, and other actions. The mHealth structure involves various activities such as diagnosis, feedback, query response, data entry, data updates, and development of smart apps, among other actions. In the context of a gig economy, these activities can be simplified to smaller tasks or gigs that can be carried out or shared among several independent contractors or freelancers.

## Methods

To carry out this research on the integration of mHealth systems in the context of the gig economy, a review and systematic search of articles, books, and opinions that allowed answering the research questions were executed through the internet. Only bibliographic sources that presented serious, reliable, and relevant information on the subject of research were included and analyzed.

In this search, the concept and characteristics of the gig economy, advantages and disadvantages, models, and examples of this economy were investigated. Similarly, the general features of the mHealth technologies and the possibility of integrating them into the gig economy were examined. In addition, some cases of health care systems in which some ideas related to the gig economy have been applied were considered. Similarly, the role played by the internet and mobile apps in the development of the gig economy and mHealth technologies was explored.

## Results

**Figure 1:**
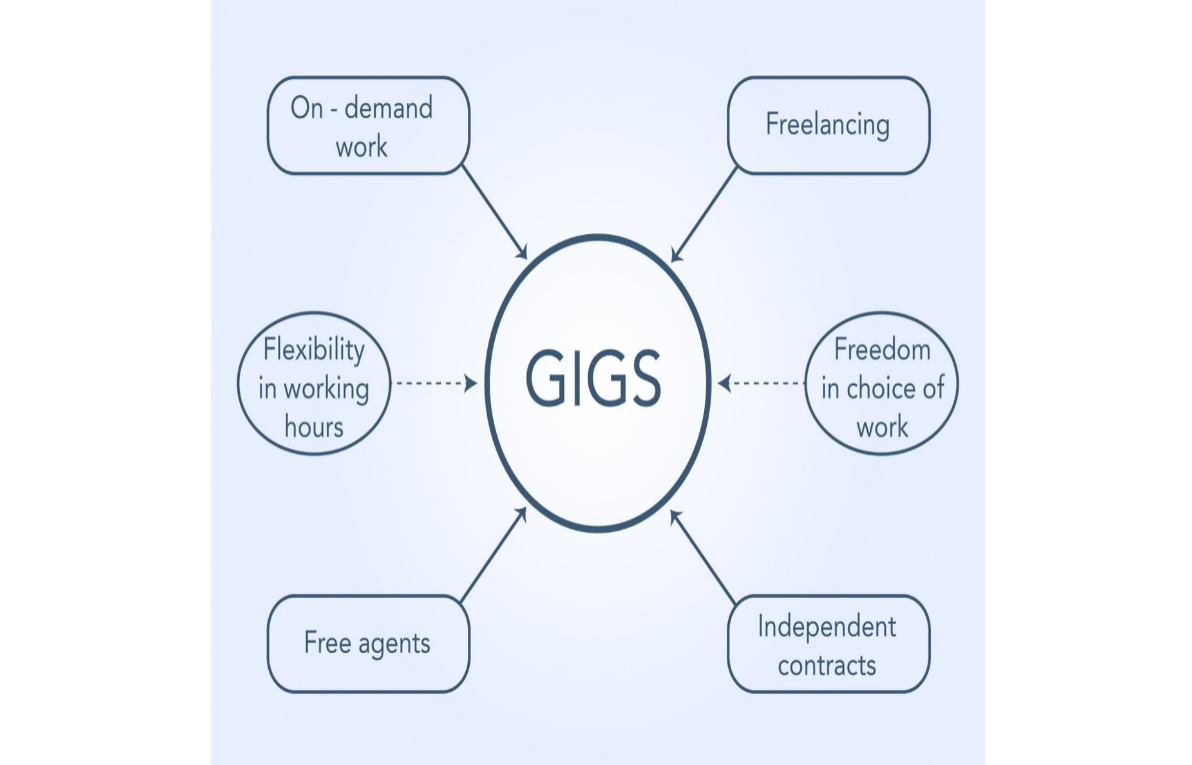
Gig economy structure.

An overview of a gig economy structure is presented in Figure 1. This figure suggests that the characteristics of a gig economy consider flexibility in working hours, on-demand work, free agents, freelancing, freedom in the choice of work, and independent contracts.

**Figure 2:**
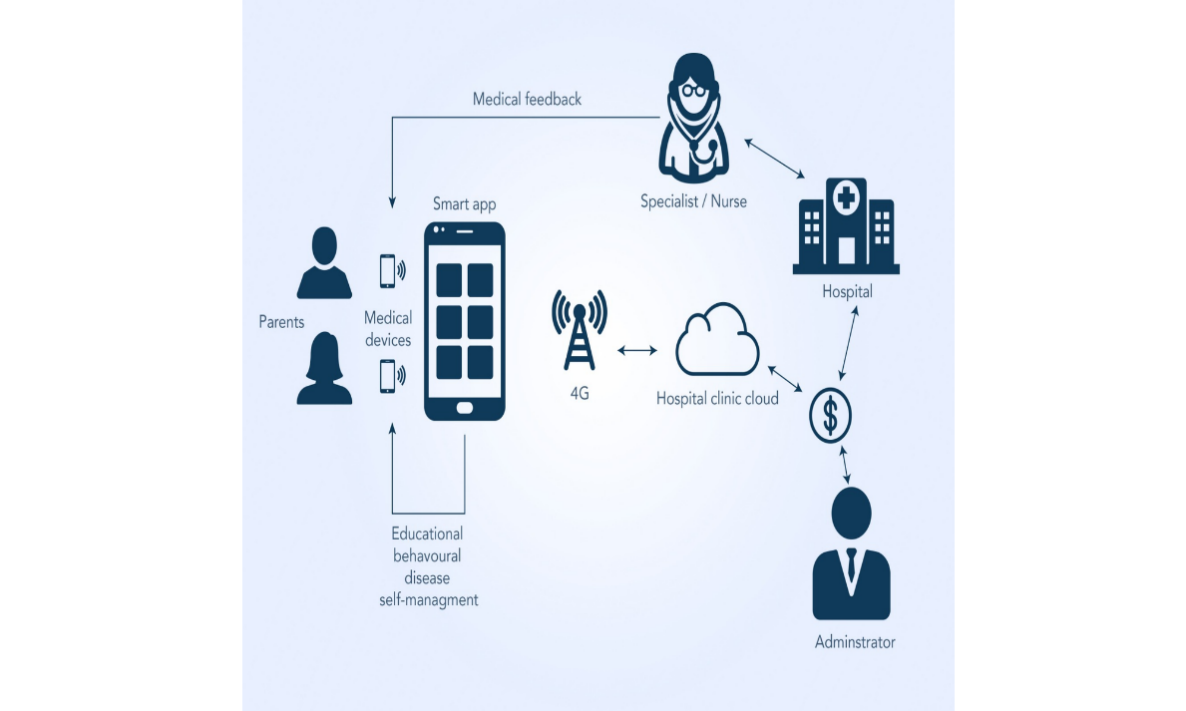
General mobile health framework.

Similarly, Figure 2 shows a common mHealth structure integrated by patients, specialists/nurses, and database administrators [11]. The patients and specialists/nurses are connected to the cloud services for the transmission of data and information. According to the figure, the patients manage the disease themselves remotely using a mobile app connected to the cloud services. Analogously, the specialists and nurses linked to these services on the cloud analyze the received data from the patients and send medical information to them based on the diagnosis results and their questions. Here, the administrators update the database and app features, among other technical tasks.

**Figure 3:**
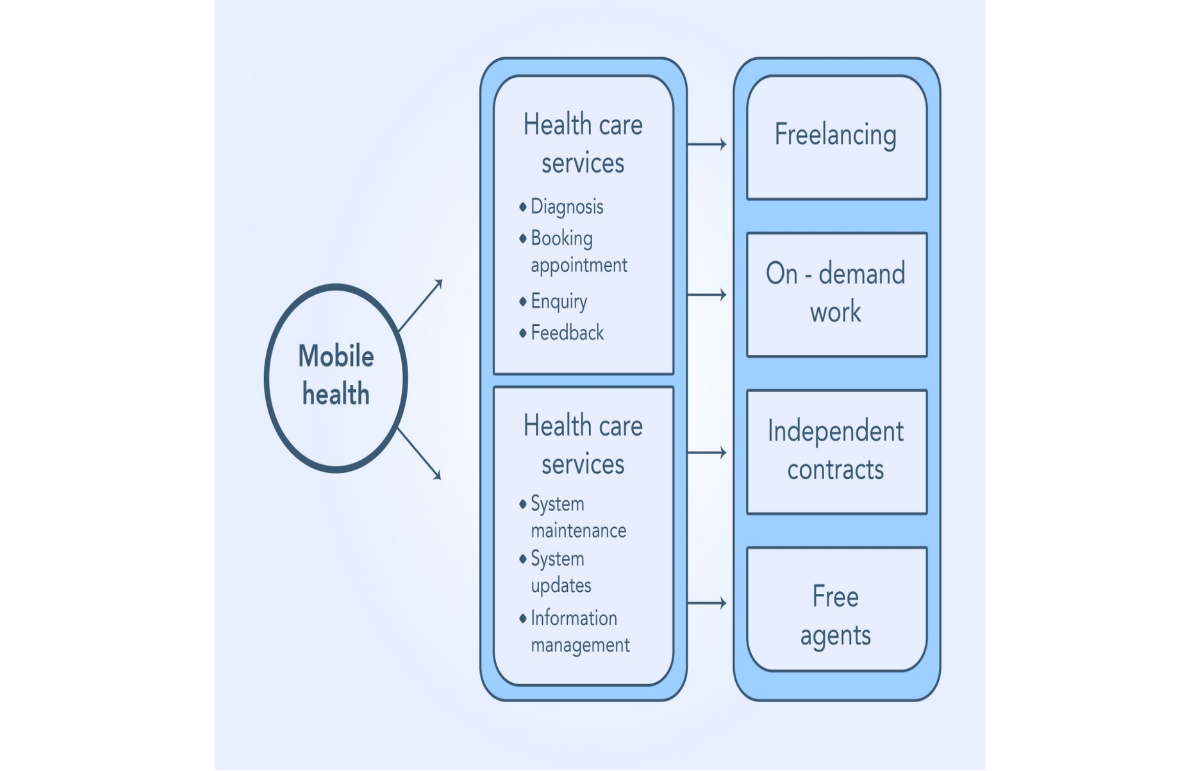
General gig mobile health system.

On the contrary, a general structure of an integrated gig mHealth system is shown in Figure 3. In this figure, the mHealth care services and the mHealth care activities are incorporated into a gig economy system categorized by on-demand work, free agents, independent contracts, and freelancing.

**Figure 4:**
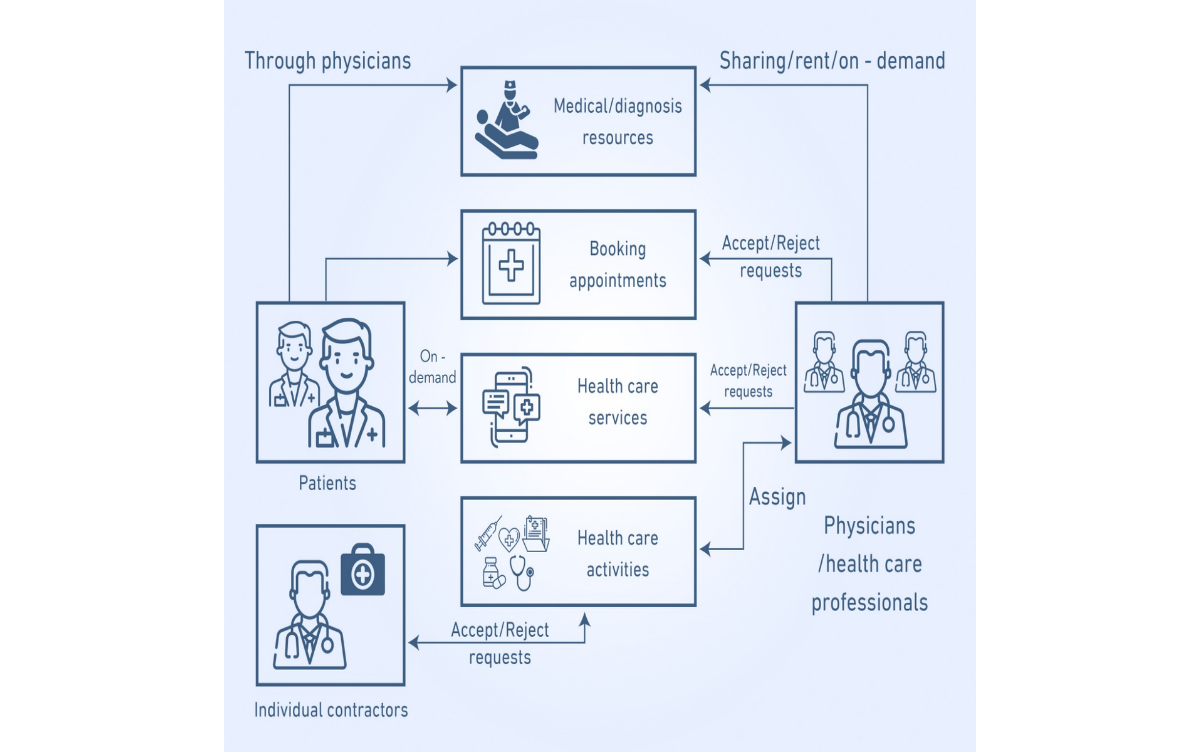
Gig economy adoption in a mobile health framework.

In addition, a practical example of an integrated view of a gig economy app in mHealth is presented in Figure 4. This figure illustrates the interaction between the patients (consumers) and the providers (partners) of mHealth care services, mHealth care activities, health care professionals, and individual contractors.

## Discussion

### Principal Findings

This research proposes the application of the gig economy in mHealth systems, which gives rise to a new dimension for health care services and to a new term that we have called *gig mHealth*. The gig/sharing economy can be applied in mHealth systems for effective use of resources such as medical equipment, physicians, nurses, diagnosis, and access to health care services in general. A gig mHealth dimension can be defined as “an approach of integrating the gig economy/sharing concepts such as freelancing, independent contracts, crowdwork, and on-demand works into the m-health framework, mainly in the healthcare services and activities.” This integration results in the effective management of health care activities and the delivery of health care services in an mHealth framework.

As shown in Figure 3, in a general gig mHealth system, the mHealth care services (diagnosis, booking appointments, enquiry, and feedback) and the mHealth care activities (system maintenance, system updates, and information management) are integrated into a gig economy system characterized by freelancing, on-demand work, independent contracts, and free agents. In this model, the mHealth care activities and the mHealth care services can be simplified to smaller tasks (gigs), and these gigs can be outsourced or shared among many independent contractors.

To integrate the mHealth systems in a practical way with the characteristics of a gig economy similar to Uber, Upwork, Turo, and other companies, it is convenient to follow the guidelines of the so-called aggregator business model that encompasses the general principles on which the economic structures of the mentioned companies are based. This model is also called the on-demand delivery model or the Uber for X model [41-43].

According to this model, the aggregator is a firm, company, or brand such as those mentioned previously or a company that integrates mHealth systems and the gig economy, as in our particular case, which organizes or aggregates a team of suppliers or partners to provide goods/services, gigs, to a group of clients or consumers [41-43]. The provider of goods/services, gigs, or partners sign a contract with the company that specifies the conditions of service, quality, price, and commission received by the aggregator or the organizing company. It is specified that the partners may accept or reject the offer of the aggregator [42]. The contract also specifies that the company or the firm will allow the partners to contract the service through an app available on mobile phones or in a Web-based platform [41]. Similarly, the clients, who in our case are patients, will also, use the platform or mobile phones to access the services, gigs, provided by the partners of the company. It is pertinent to indicate that the aggregator or the company helps suppliers to market their services, but they are not their employees, they are partners of the business. In this model, both consumers of services and suppliers of goods/services are customers of the company in a win-win way [41]. After a service is completed, the customer or client can rate the service and the provider, and the provider can rate the customer [42].

Following the described aggregator model, an integrated view of a gig economy app in mHealth is presented in Figure 4. As shown in Figure 2, the mHealth system already has a network of physicians/nurses, health care units, database administrators, internet connections, and mobile apps connected to the hospital/clinic cloud and patients. In addition, the mHealth structure involves various activities such as diagnosis, feedback, query response, data entry, data updates, and development of smart apps, among other actions. In the framework of a gig economy, these activities can be simplified to smaller tasks or gigs that can be carried out or shared among several independent contractors or freelancers.

In this context, as shown in Figure 4, according to the aggregator model, the consumers are the patients, and the partners are the providers of mHealth care services (diagnosis, booking appointments, inquiry, and feedback), mHealth care activities (system maintenance, system updates, and information management), health care professionals (physicians, nurses, technicians, and other staff), and individual contractors. In this model, the customers and providers are interconnected with the aggregator firm, brand, or company through digital means by using a smartphone app or Windows platforms. As suggested in the figure, the providers can accept or reject the requests of the aggregator.

In addition, in Figure 4, the scope of the gig economy app in mHealth is explained considering an integrated view of the following main areas: sharing/renting medical and diagnostic equipment and resources, on-demand appointments/self-health management, on-demand health care services, and assigning health care activities/gigs to individual contractors.

In this sense, in a gig mHealth system, it is possible to share medical and diagnostic equipment and resources to make optimal use of expensive resources such as magnetic resonance imaging scanners, positron emission tomography scanners, x-ray/computerized tomography machines, and other types of equipment that are only available in large hospitals or health care centers by renting them for a certain time when necessary. This allows sharing such equipment among the network of doctors on an hourly rental basis. This possibility benefits the access of the private doctors to the diagnostic equipment when it is required, which minimizes the costs and references and improves the speed of the provision of medical care services.

Similarly, on-demand appointments/self-health management and the freedom of choosing doctors by the patients can be a major development in health care sectors that can be reached in a gig mHealth system. This allows having access to doctors at the right time and place, which is an important aspect in the delivery of health care services. In a gig mHealth system, the doctors can register on a gig economy platform, and the registered patients can access the doctor’s/physician’s profiles and book an appointment within minutes. This can enable the freedom of access to health care for the patients 24 hours a day and 7 days a week in any place. The platform can be embedded with various features by adopting the technology. For example, if the users are not sure whether to visit a doctor, instant health advice can be provided through the app using a chat feature. Similarly, the users can create their digital twin on the app recording their current health condition and then access their predicted future health by the app.

Analogous to booking appointments, the users can select and book for on-demand health care services delivered at home such as dispensing medicines, physiotherapy, blood sample collection, glucose monitoring, and other services from various providers, which not only helps the patients in minimizing the costs of hospital visits but also lets them select the professionals and services based on their requirements.

In addition, various health care activities can be outsourced to individual contractors. For example, health care tasks such as vaccination can be assigned to nurses on a contractual basis where the children/patient information can be accessed from the mHealth systems and which can be updated after completing the jobs. There is even scope for outsourcing complex tasks such as major surgeries/operations that need to be performed by a health care expert. The professional experts in the mHealth system can accept/reject such proposals after examining the condition of the patients.

Among all the areas of apps examined, reviews and ratings can be used as tools to measure the abilities and capabilities of the resources. In any case, the major point of the application of the gig economy in mHealth is to simplify the health care activities/tasks into gigs/small pieces of work that can be assigned to individual contractors.

In relation to the previous ideas, it is worth mentioning that there are some examples of applications of the gig economy in the health care area. One of them is Nomad, which is a Web-based platform that connects freelance doctors and nurses to hospitals directly [7]. Through this platform, doctors and nurses negotiate their contracts, gigs, with hospitals. Nomad charges a commission for facilitating this connection. Other similar platforms are Enzyme and Medely [8,9].

As the possibility of integrating mHealth within a gig economy model represents a new technical framework that can provide benefits in health care delivery, it is necessary to carry out more research to investigate the impact of integrating mHealth real cases in the structure of the gig economy.

### Conclusions

The analysis carried out in this study suggests the possibility of integrating mHealth within the framework of the gig economy, enhancing health care services delivery and the management of health care activities. The following 4 major areas of applications proposed in the mHealth framework that can catalyze the operations using the features of the gig economy are sharing/renting medical and diagnostic equipment and resources, on-demand appointments/self-health management, on-demand health care services, and assigning health care activities/gigs to individual contractors. This integration leads to a new dimension for health care services and the proposal of a new term: gig mHealth.

## Abbreviations

mHealth: mobile health
